# Contraception among bankers in an urban community in Lagos state, Nigeria

**DOI:** 10.11604/pamj.2013.14.80.2216

**Published:** 2013-02-27

**Authors:** Ijeoma Angela Meka, Emmanuel Chidiebere Okwara, Anthony Obiamaka Meka

**Affiliations:** 1 Department of Chemical Pathology, University of Nigeria Teaching Hospital, Ituku-Ozalla, Enugu State, Nigeria; 2 Department of Chemical Pathology, Imo State University Teaching Hospital, Orlu, Imo State, Nigeria; 3 German Leprosy and Tuberculosis Relief Association, Independence Layout, Enugu, Nigeria

**Keywords:** Contraception, bankers, awareness, knowledge, practice, prevalence

## Abstract

**Introduction:**

Contraception means procedures employed to interfere at one stage or the other with the normal sequence of events in the process of reproduction leading to a failure in conception. It means voluntary techniques adopted to achieve birth control. Its use remains sensitive worldwide. Within the same society, contraception varies amongst people of different socio-cultural, educational, religious, or occupational affiliations. It also varies between urban and rural settlements. Some contraceptive techniques also prevent sexually transmitted infections (STIs). The prevalence of STIs also varies with these same factors. There is very limited literature on contraception exclusively amongst bankers. We sought to investigate the level of awareness and practice of contraception amongst bankers in an urban society in Lagos State, Nigeria.

**Methods:**

In this descriptive cross-sectional study, 200 self-administered structured questionnaires were retrieved from bankers from 5 banks selected by simple random sampling in Surulere Local Government Area of Lagos State, Nigeria. Data was subsequently statistically analyzed using SPSS.

**Results:**

The age range was 21-45 years, mean28.8±1.4 years, 51.7% were males (72% single, 27% married, 1% separated) and 48.3% were females (52.4% single, 47.6% married). All (100%) respondents were aware of contraception, 93.3% males and 91.7% females were sexually active, 88.9% males and 84.5% females believe contraception is useful. Most (71.4%) respondents practice contraception, males (81%) being more than females (61.1%), p < 0.05. More (71.4%) females and fewer (37.8%) males believe that contraception prevents pregnancy but not STIs, 28.6% of females and 46.6% of males believe it prevents both pregnancy and STIs, whereas 14% of males and no female believe contraception prevents STIs but not pregnancy.

**Conclusion:**

The awareness of and practice of contraception was very high among the bankers but more male bankers practice contraception whereas more female bankers perceive contraceptives to be for the married only.

## Introduction

Contraception means procedures employed to interfere at one stage or the other with the normal sequence of events in the process of reproduction leading to a failure in conception [1]. It can also be defined as the intentional prevention of conception or impregnation through the use of various devices, agents, drugs, sexual practices or surgical procedures [2]. It is any voluntary technique adopted to achieve birth control. Its use remains sensitive worldwide. Within the same society, adoption of contraception varies amongst people of different socio-cultural, educational, religious, or occupational affiliations [3–5]. It also varies between urban and rural settlements [5–8]. Some contraceptive techniques (barrier methods) also prevent sexually transmitted infections (STIs) while extensive use of emergency contraception (EC) may increase risk for contacting STIs [9]. The prevalence of STIs also varies with these same factors [10]. The level of awareness of contraception is high in most developing countries but the practice of any form of contraception remains low in these countries [10, 11]. The contraceptive prevalence (percentage of women 15-49 years who are practicing or whose sexual partners are practicing any form of contraception) varies widely across nations [10]. In 2008 it was 8% in Sierra Leone, 15% in Nigeria, 16% in Mozambique, 24% in Ghana whereas it was 69% in Netherlands, 79% in United States and Paraguay and 82% in United Kingdom [10]. In a study carried out in Nnewi, Anambra State, South-East Nigeria, whereas awareness of contraception was 80% and acceptance was 87% prevalence rate was only 25% even though 90% of respondents were literate [11]. In a North-Eastern Nigeria rural population, the awareness of rhythm method, lactational amenorrhoea method and coitus interruptus was 50.7%, 42.1% and 36.1%, respectively and even lower for modern forms of contraception [12]. Bankers represent a population of highly professional and informed individuals whose choice of career will influence their general attitudes and practices including contraception. The prevalence of contraception amongst these professionals may vary widely with that of the general population and with that of other professionals. The low prevalence of contraception [10, 11] observed in Nigeria in the general population may therefore vary widely from that of bankers. We sought to investigate the level of awareness and practice of contraception amongst bankers in an urban population in Lagos State, South-Western Nigeria.

## Methods

In this descriptive cross sectional study, structured self-administered questionnaires were given to 200 bankers (comprising 103 males and 97 females) in Surulere Local Government Area of Lagos State, Nigeria. This local government is located in Lagos mainland in Lagos state with an area of 23 km^2^, a population of 503,975 inhabitants (2006 census) with a population density of 21,864 inhabitants per km^2^ [13]. Five banks were selected in the local government by simple random sampling, 40 questionnaires was administered to respondents in each bank. Only willing respondents were included. In each of the selected banks, permission was obtained from the personnel manager or head of operations. On practice of contraception, respondents were to answer in the affirmative to any question if either they or their sexual partners practice the method of contraception concerned. Each questionnaire was retrieved immediately after completion. All administered questionnaires were retrieved. Subsequently analysis was done using SPSS, p-values less than 0.05 were regarded as statistically significant.

## Results

The age range of respondents was 21-45 years with a mean age of 28.8±1.4 years, 51.7% were males (72% single, 27% married, 1% separated) and 48.3% were females (52.4% single, 47.6% married).

### Knowledge

All (100%) respondents were aware of contraception. Awareness of condom was the highest (63%) followed by pills (47%), safe period (41.5%), coitus interruptus (36.7%), abstinence (35.6%), Billing's method (23.3%), diaphragm (20.1%), vasectomy (20.1%), intrauterine contraceptive device (IUCD) (19.8%), injectables (17.3%), implants (16.2%) and spermicides (16.1%) whereas awareness of tubal ligation was the least (14.9%) (Table 1).

**Table 1:** Knowledge of contraception among bankers

Method of contraception	Percentage
Condom	63
Pills	47
Safe Period	41.5
Coitus interruptus	36.7
Abstinence	35.6
Billing's method	23.3
Diaphragm	20.1
Vasectomy	20.1
IUCD	19.8
Injectables	17.3
Implants	16.2
Spermicides	16.1
Tubal ligation	14.9
Any one method	100

More (71.4%) females than males and fewer (37.8%) males believe that some methods of contraception prevent pregnancy but not STIs (p < 0.05). More males (46.6%) than females (28.6%) believe that some methods of contraception prevent both pregnancy and STIs (p < 0.05), whereas 14% of males and no female wrongly believe that contraception prevents STIs but not pregnancy.

### Attitude

Most respondents (93.3%) males and (91.7%) females were sexually active. Most respondents also believe contraception is useful (approval of contraception), of which there were more males (88.9%) and fewer females (84.5%) but this difference in approval of contraception is not statistically significant (p >0.05) between both gender.

### Practice

Most respondents (71.4%) practice at least one method of contraception, males (81%) significantly more than females (61.1%), p < 0.05. For those who practice contraception, condom was the commonest method (70% males, 66% females), followed by safe period method (38.2% males, 36.2% females), penile withdrawal method (32.4% males, 14.9% females), pills (8.8% males, 14.9% females), injectables (5.9% males, no female), implants (no female banker), 2.9% males), intrauterine contraceptive device (6.4% females, no males) (Table 2).

**Table 2:** Practice of contraception among bankers

Method of contraception	Males	Females
Condom	70	66
Safe Period	38.2	36.2
Coitus interruptus	32.4	14.9
Pills	8.8	14.9
Injectables	5.9	0
Implants	2.9	0
IUCD	0	6.4

## Discussion

Over 90% of the bankers in our study population are sexually active and all are aware of at least a method of contraception with nearly 90% approval of contraception but much lower (71.1%) prevalence of contraception. This value of prevalence of contraception in our study population is however very much higher than were observed in general population in other urban town studies in Nigeria [11, 14] and also very much higher than the reported national prevalence [10] as well as prevalence in selected rural locations in Nigeria [15]. It is even higher than the general population prevalence in some developed countries and as high as in other developed countries [10]. This might be attributed to their level of education and influence of their work environment. In this study, we observed that significantly more male bankers (or their sexual partners) practice contraception than the female bankers (or their sexual partners). One would have expected the reverse to be the observation since women carry the burden of pregnancy. This observation may also not be unconnected with the African culture which generally leaves women with low bargaining power in reproductive issues. We also observed that significantly more female bankers wrongly believe that contraception is for the married only and this may have accounted for the lower practice observed in this gender. This is contrary to expectation giving their educational status but religion and culture may have roles to play in this regard.

Although prevalence of contraception was observed to be very high in this population the use of the more reliable and reversible methods *vis a vis* implants, pills, injectables, IUCD enjoyed very low patronage. A very high usage was observed for condoms. This may be due to its additional benefit of preventing STIs, which implants, pills and IUCD does not possess and the belief of having no side effects.

## Conclusion

The awareness of and practice of contraception was seen to be very high among bankers in Surulere, Lagos, Nigeria but significantly more male bankers practice contraception whereas significantly more female bankers believe contraceptives is for the married only.

### Limitations

Unwillingness of some staff to participate in the study was a major constraint in the study.
